# First Documented Resighting of a Dwarf Minke Whale From Calf to Adulthood in the Great Barrier Reef Indicates Long‐Term Colour Pattern Stability and Site Fidelity

**DOI:** 10.1002/ece3.73851

**Published:** 2026-06-18

**Authors:** Claire E. Wouters, R. Alastair Birtles, Suzanne Hillcoat, Naomi M. Gardiner

**Affiliations:** ^1^ College of Science and Engineering James Cook University Townsville Queensland Australia; ^2^ Minke Whale Project James Cook University Townsville Queensland Australia

**Keywords:** cetaceans, conservation, ecology, environmental monitoring, great barrier reef, protected areas, recruitment

## Abstract

Dwarf minke whales occur throughout the Southern Hemisphere but the only predictable winter aggregation area is in the northern Great Barrier Reef (GBR). Interannual site fidelity has been recorded frequently there for > 20 years in males and breeding females; mothers with calves are seen occasionally, but a calf has never been recorded as returning after its birth year. Every photographed calf between 2003 and 2023 was compared with a catalogue of known GBR individuals and MW0088 ‘Rudi,’ first seen as a calf in 2006, was found to have been resighted intermittently to 2017—when presumably a mature 11‐year‐old. This is the first direct evidence for natal wintering ground fidelity in GBR dwarf minke whales and provides one of the longest sighting histories in the GBR and globally. Detailed analysis of MW0088's colour patterns showed almost no variation over the 12‐year period, confirming their value for individual photo‐identification over extended periods. The likely repeated long‐distance migrations of MW0088 to the same GBR location support the designation of this aggregation area as biologically important for this population of a still undescribed minke whale subspecies.

## Graphical abstract

A predictable winter aggregation of dwarf minke whales occurs annually in the Great Barrier Reef, where interannual site fidelity has been documented in adults but not previously in calves. Comparison of all photographed calves from 2003 to 2023 with a long‐term photo‐identification catalogue revealed one individual resighted from calf to adulthood, providing evidence of natal wintering ground fidelity and temporal stability of colour patterns. These findings support the ecological significance of the northern Great Barrier Reef for this yet‐undescribed subspecies.

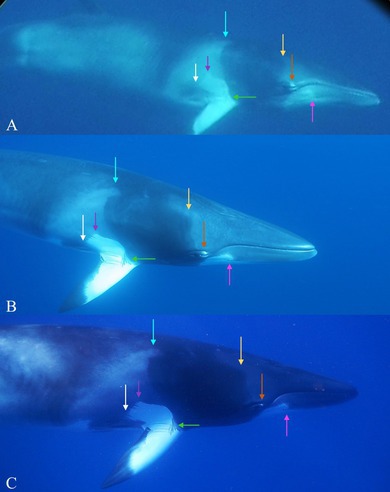

## 1 Introduction

Dwarf minke whales ( *Balaenoptera acutorostrata* ) are seasonal migrants in east Australian waters, with a documented migration path from a well‐established aggregation area in the northern Great Barrier Reef (GBR) during austral winter (May through August) to feeding grounds in the vicinity of the Antarctic convergence (Birtles et al. 2015; Kato et al. 2021). Sightings in this wintering ground are concentrated on the outer shelf to the west of Ribbon Reef No. 10, but have been reported from the Agincourt Reef complex (southernmost area 15°14'S) to the cross‐shelf reefs of Waining, Parke and Jewel Reefs in the north (at approximately 14°24'S) (Birtles et al. 2008; Curnock 2010; Sobtzick 2010). This region represents the only known predictable aggregation of dwarf minke whales in the world (Arnold and Birtles 1999). The inquisitive behaviour of these aggregating whales (Mangott 2010; Birtles et al. 2001, 2014) facilitates a regulated swim‐with‐dwarf minke whales ecotourism industry (Arnold and Birtles 1999; Birtles et al. 2002) that also enables data collection and photo‐identification of individual whales. All life stages, including juveniles, adults and females with calves, are observed and the sex of interacting whales is occasionally identified through whale behaviours revealing their ventral surface, like belly presentations (Arnold 1997; Birtles et al. 2001, 2014; Dunstan et al. 2007; Sobtzick 2010).

GBR dwarf minke whale sighting reports only became mandatory in 2003 and have revealed that less than 5% of encounters include mother‐calf pairs every year (Birtles et al. 2002, 2014; Dunstan et al. 2007). Under the Australian National Guidelines for Whale and Dolphin Watching (Commonwealth of Australia 2017) and the Code of Practice for dwarf minke whale interactions in the Great Barrier Reef (Birtles et al. 2008) for swim‐with permit holders, a vessel cannot approach closer than 300 m to a calf and entering the water to swim with them is prohibited. As a result, identifiable imagery of mothers and calves is restricted to encounters where the whales approach swimmers already in the water and these are usually brief as the mother is often wary and typically less interactive than other whales (Birtles et al. 2002, 2008). Nevertheless, this scarce imagery has proved valuable, as identifying individual resighting histories is crucial for establishing effective management strategies and understanding the function and significance of this aggregation. For example, a long‐term photo‐identification study has recently led to the expansion of the southern right whale reproduction Biologically Important Area (BIA) into southwestern Australia (Salgado Kent et al. 2022).

Photo‐identification of individual dwarf minke whales is completed using multiple colour pattern elements along the length of the body, particularly around the shoulder area (Arnold et al. 2005; Sobtzick 2010; Hutchings et al. 2023). This method has previously confirmed extensive interannual site fidelity to the area around Ribbon Reef 10 in the GBR (Birtles et al. 2001, 2002; Sobtzick 2010; Barr 2023). Despite this, no calf has ever been resighted after its birth year in the GBR or globally, leaving it unknown what changes, if any, may occur to colour patterns in the first few years of life until sexual maturity, which is estimated to be between seven and 10 years of age (Kato et al. 2021). Here, we report the first record of a dwarf minke whale calf resighted after its birth year and documented from calf to adulthood through photo‐identification, with sightings spanning a 12‐year period. This represents the first direct evidence for natal wintering ground fidelity in the GBR and of probable natal philopatry.

## 2 Materials and Methods

A catalogue of nearly 600 individual dwarf minke whales seen between 2006 and 2008 and 2017 to 2019 has been compiled and managed by The Minke Whale Project (MWP; www.minkewhaleproject.org), which collects thousands of images and videos annually via hand‐held underwater cameras by researchers, or donated by passengers and crew onboard swim‐with tourism vessels (Sobtzick 2010; Birtles et al. 2014). Nine active swim‐with whale permits are used annually for the northern Great Barrier Reef, each following a Code of Conduct (Birtles et al. 2008). Each sighting was assigned a unique Encounter ID number and the total number of whales in the swim‐with encounter was estimated using the maximum number of whales seen at one time. Individual whales were identified from available imagery using colour patterns and visible scarring, a methodology outlined in Sobtzick (2010). The illegal nature of entering the water with mother‐calf pairs resulted in only 76 recorded encounters between 2003 and 2023 where identifiable imagery of a calf was obtained (Barr 2023), those being when a mother‐calf pair passed close to swimmers already in the water. This calf imagery was systematically checked against the main catalogue and new individuals were incorporated as unique whales. The minimum duration of an individual's interaction with a vessel was estimated using the time elapsed between the first and last photograph of the whale taken during each encounter, using image timestamp metadata from images.

## 3 Results

**FIGURE 1:**
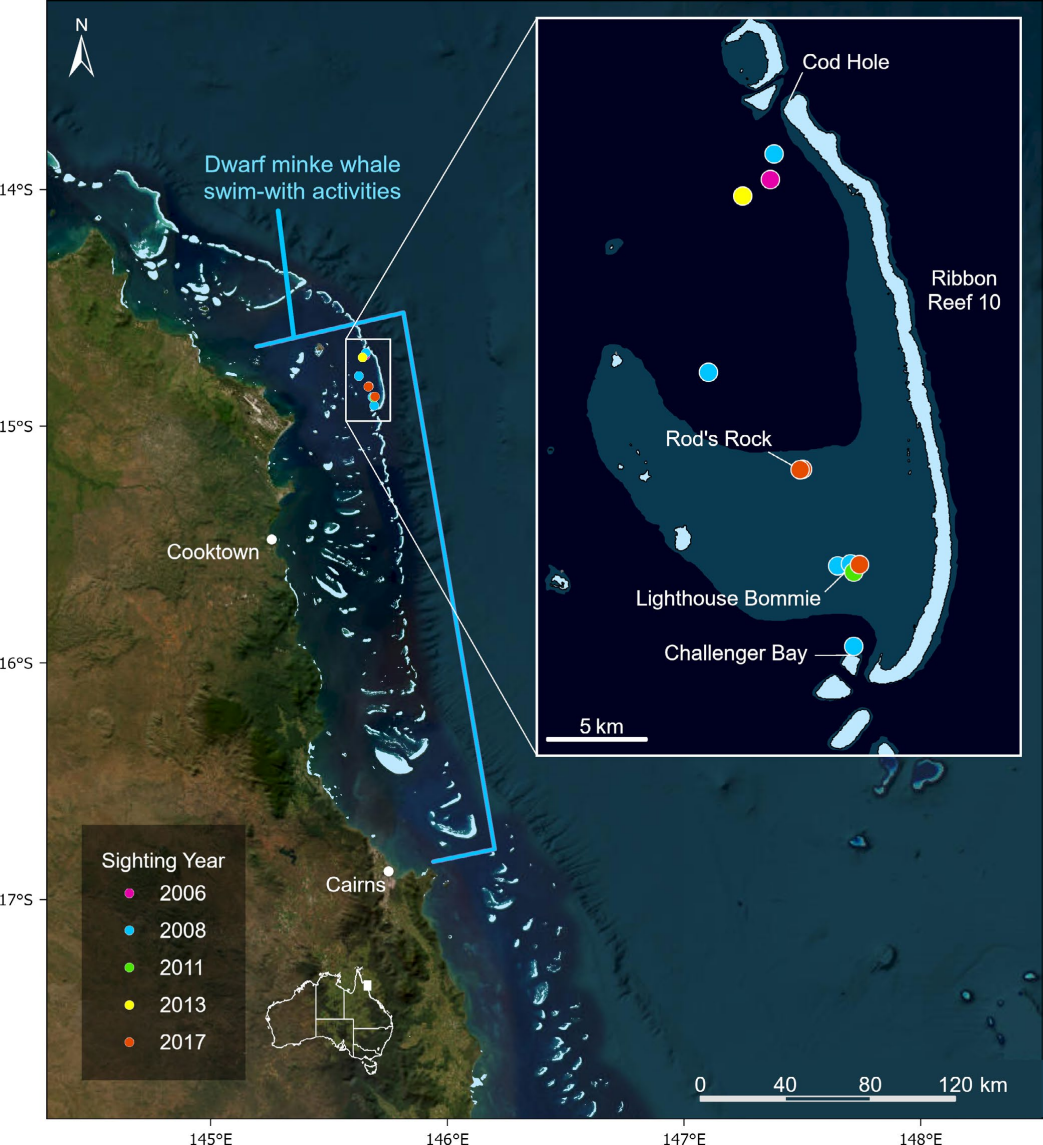
Spatial distribution of dwarf minke whale MW0088 ‘Rudi’ sightings.

A dwarf minke whale calf (ID code MW0088, ‘Rudi’) was observed on 6 July 2006 near the top of Ribbon Reef No. 10 in the GBR (Figure 1), accompanied by a large female dwarf minke whale (ID code MW0270) presumed to be its mother. The mother‐calf pair approached swimmers multiple times, and ‘Rudi’ was often swimming independently, allowing imagery from the left and right side to be documented.

MW0088 ‘Rudi’ was next sighted in five encounters in 2008 as a two‐year‐old, in the same area off Ribbon Reef No. 10 (Figure 1, Table 1). Rudi's mother was also next observed in 2008 and in the same area, but around a month earlier, on 16 and 18 June. ‘Rudi’ continued to return to this area for multiple years afterwards (Table 1) until the last sighting in 2017 as an 11‐year‐old adult. Across all years, the maximum distance between sighting locations was 16 km.

**TABLE 1:** Sighting details for dwarf minke whale ID code MW0088 ‘Rudi’. Sighting interval refers to the interval (days) between first and last sighting for the respective year. Total whales in encounter refers to the reported number of whales seen at one time during an encounter.

Year (interval)	Encounter Number	Date	Location	Nearest reef	Total whales in encounter
2006 (1 d.)	UE.06.07.06.1	06‐Jul‐2006	14°42′S, 145°′E	Cod Hole	2
2008 (10 d.)	UE.08.07.14.2	14‐Jul‐2008	14°53′S, 145°41′ E	Lighthouse Bommie	9
	UE.08.07.16.2	16‐Jul‐2008	14°53′S, 145°41′ E	Lighthouse Bommie	8
	UE.08.07.22.1	22‐Jul‐2008	14°54′S, 145°41′ E	Challenger Bay	13
	UE.08.07.22.2	22‐Jul‐2008	14°47′S, 145°38′ E	Ribbon 10	2
	TA.08.07.23.1	23‐Jul‐2008	14°41′S, 145°39′ E	Cod Hole	2
2011 (1 d.)	EE2.11.07.10.3	10‐Jul‐2011	14°53′S, 145°41′ E	Lighthouse Bommie	9+
2013 (1 d.)	UE.13.06.24.1	24‐Jun‐2013	14°42′S, 145°38′ E	Ribbon 10	8
2017 (11 d.)	SF.17.06.28.2	28‐Jun‐2017	14°50′S, 145°40′ E	Rod's Rock	8
	SF.17.07.05.3	05‐Jul‐2017	14°50′S, 145°40′ E	Rod's Rock	6
	SS.17.07.08.1	08‐Jul‐2017	14°53′S, 145°41′ E	Lighthouse Bommie	12

Throughout the 12‐year sighting history (Table 1), ‘Rudi’ was interactive with swimmers, allowing identifiable photographs to be collected of their colour pattern profile. In the 2011 and 2013 encounters, author R.A.B. observed ‘Rudi’ intermittently for over 3 h and recorded at least six close passes during each encounter ranging from 1.0 to 8.0 m. When seen in encounter SS.17.07.08.1 (Table 1) in 2017, ‘Rudi’ again remained in close proximity to swimmers for at least 4 h, making both distant and close approaches. Unfortunately, belly presentations, submerged tail stands or head rise behaviour that would allow a sex identification were not photographed in any of these encounters, so ‘Rudi's sex is still unknown. In the last 2017 sighting, ‘Rudi’ was swimming in proximity to several assumed sexually mature females. However, insufficient information about dwarf minke whale courting behaviour precludes a sex identification based on group composition alone.

**FIGURE 2:**
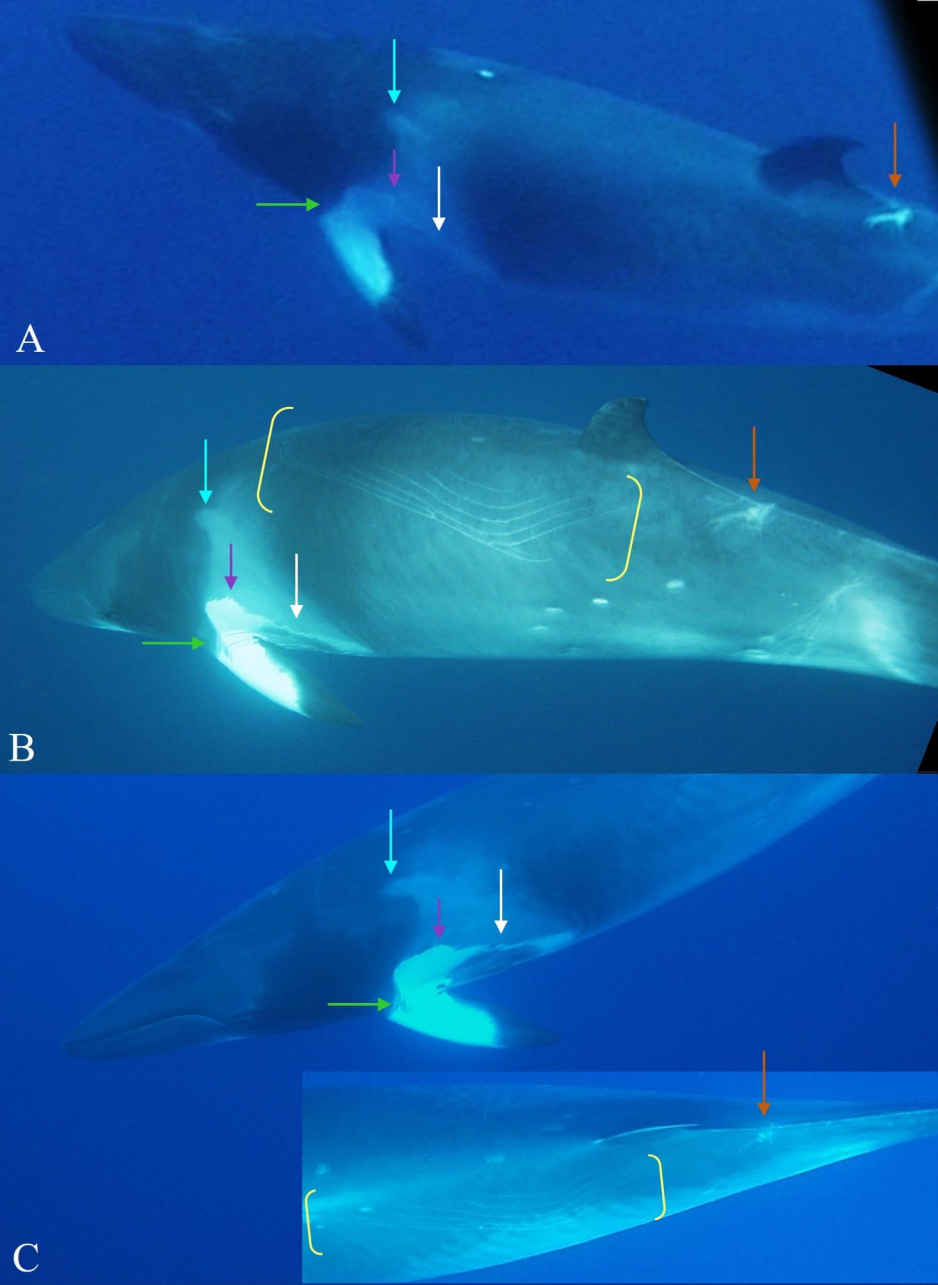
Stability of the left‐side colour pattern profile and scar prevalence of dwarf minke whale MW0088 ‘Rudi’ through time. (A) ‘Rudi’ as a calf on 6 July, 2006 (image from video by Susan Sobtzick). (B) At age 2 y. on 14 July, 2008 (image by Alastair Birtles). (C) At age 11 y. on 28 June, 2017 (photos by Emily Daley). The coloured arrows compare identifying marks used to confirm that it is the same animal. Scarring visible in 2006 (A) was still identifiable in 2008 (B) (orange arrow), and a large V‐shaped rake scar on the left flank (yellow bracket) first documented in 2008 (B) was still present in 2017 (C).

**FIGURE 3:**
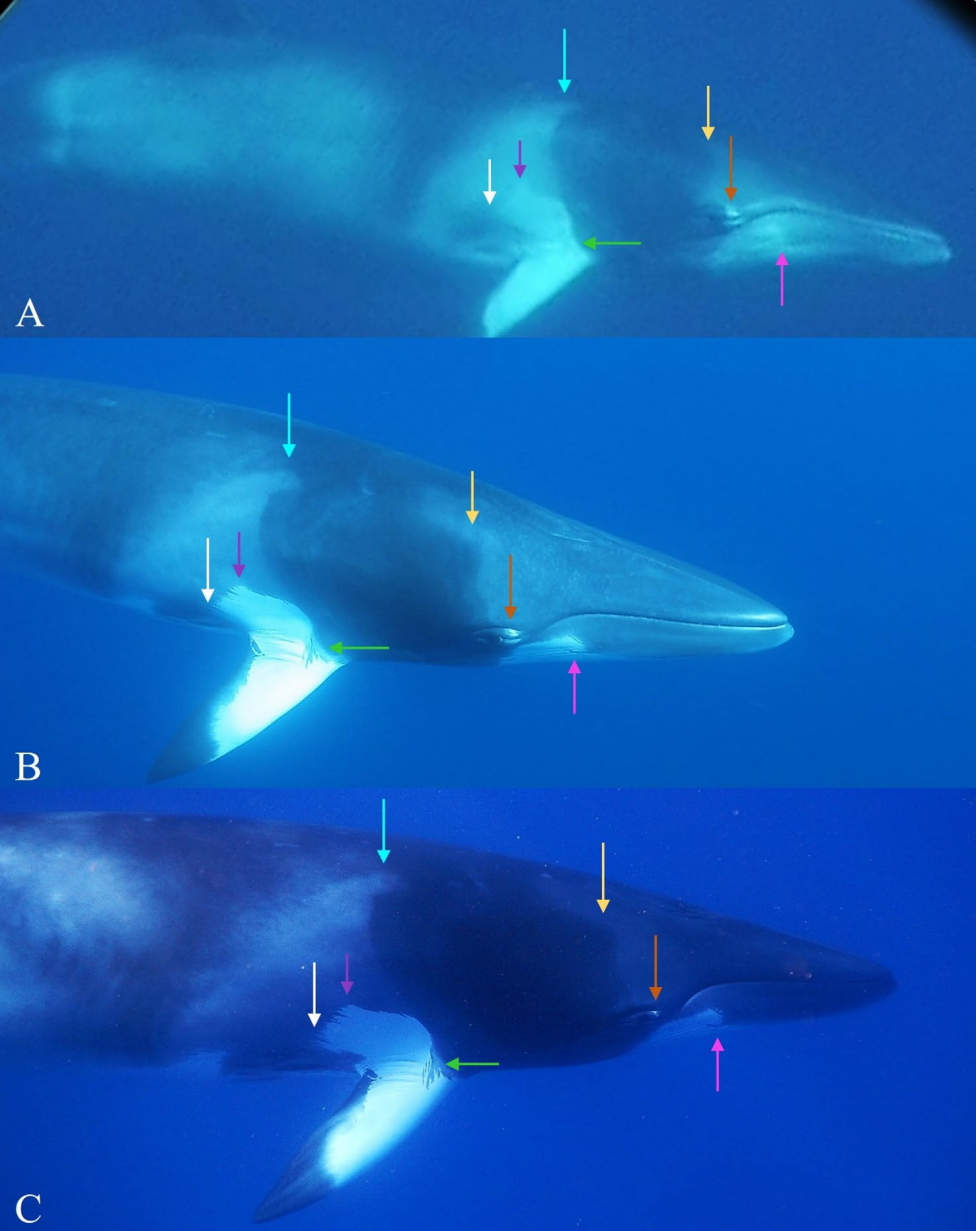
Stability of dwarf minke whale right‐side colour pattern profile during maturation. A: MW0088 ‘Rudi’ as a calf on 6 July, 2006 (image from video by Susan Sobtzick). B: At age 2 y. on 14 July, 2008 (image by Alastair Birtles). C: At age 11 y. on 28 June, 2017 (image by Alastair Birtles). The coloured arrows compare identifying marks used to confirm that it is the same animal.

Comparison of MW0088's colour pattern profile as a calf, a two‐year‐old, and an 11‐year‐old revealed that these patterns are remarkably stable throughout life (Figures 2, 3). The only visible difference was the extent of the right‐side eye blaze, which appeared to have reduced with age and become less diffuse (Figure 3A,C). The shape of all other colour elements, including the rostral saddle, white pectoral fin and shoulder blaze, light grey thorax patch, diffuse flank infill and white peduncle blaze (Arnold et al. 2005), remained stable throughout the entire sighting history. Rake scarring on the dorsal peduncle, gained prior to being seen in the GBR as a calf in 2006, was fully healed by the 2008 resighting and still visible in 2017. Similarly, the large V‐shaped rake scars first observed in 2008 along the upper left flank were still clearly visible in 2017 (Figure 2A–C), despite apparent pigmentation. Lastly, a fresh wound likely from a cookie cutter shark (*Isistius* sp.) (Arnold et al. 1987; Dwyer and Visser 2011) above the right shoulder in 2006 was still faintly visible on the 11‐year‐old in 2017.

## 4 Discussion

This observation provides the first documented resighting of a known dwarf minke whale from the neonatal stage through to presumed sexual maturity, providing the strongest evidence to date for natal wintering ground fidelity in dwarf minke whales. Documenting the return of this calf to the Great Barrier Reef (GBR) aggregation area near Ribbon Reef 10 over a 12‐year period contributes valuable detail to our understanding of dwarf minke whale behaviour, life history, and colour pattern stability. Natal philopatry is also likely, as ‘Rudi’ was 11 years old in 2017, and sexual maturity is estimated to be around the age of seven to 10 years for dwarf minke whales (Kato et al. 2021). Additionally, high levels of courtship behaviour have been documented in the GBR aggregation area (Birtles et al. 2002; Mangott 2010; Birtles et al. 2014), and the timing of calf births (Kato et al. 2021; Barr 2023) also suggests mating occurs in the GBR.

Natal wintering ground fidelity can play an essential role in shaping the genetic structure of wintering populations and highlights areas that are of high ecological value to populations (Baker et al. 2013; Carroll et al. 2016). Dwarf minke whales migrate annually from southern feeding grounds to the northern GBR, where they aggregate during the austral winter (May–August) to breed and rest (Gedamke 2004; Mangott 2010; Sobtzick 2010; Barr 2023) before migrating south along the east Australian coastline (Birtles et al. 2015). Available sighting and stranding data suggest that calves are born during the northbound migration (Arnold 1997; Barr 2023), and the close dependency of the calf on its mother during the 6‐month lactation period (Horwood 1990; Kato 1995) provides a maternally directed mechanism for learning migratory routes that the calf may use for the rest of its life.

While the natal wintering ground site fidelity and potential natal philopatry in this single‐animal observation could represent an atypical case for this population, it is more likely to reflect a common occurrence that has gone undocumented thus far due to the time‐intensive nature of individual photo‐identification of these whales, the lack of identifiable calf imagery, and the non‐systematic nature of data collection using platforms of opportunity from the swim‐with‐minke whale dive tourism industry. Inter‐annual site fidelity to the northern GBR aggregation area near Ribbon Reef 10 has previously been documented for this population using photo‐identification (Birtles et al. 2001, 2002; Sobtzick 2010; Barr 2023), however, this is the first recorded instance of an individual first observed as a calf returning in subsequent years.

The stability of pigmentation patterns in the genus *Balaenoptera* is unknown, unlike humpback whales, which have stable fluke patterns from birth (Cheeseman et al. 2023). As such, until now, there have been no definitive cases for *Balaenoptera* species where a calf has been re‐identified as an adult using photo‐identification alone. It is therefore possible that this is a unique individual and stable colour patterns from calf to adult are not the norm, such that other individuals do not get re‐identified because their patterns change with age. However, given the successful long‐term photo‐identification study of these whales in the GBR using their colour patterns alone (Birtles et al. 2001, 2002; Sobtzick 2010; Barr 2023), the lack of similar re‐sightings is more likely the result of under‐sampling the population and the very limited available calf imagery. If colour patterns are stable from calving in this undescribed subspecies then the same could occur in other *Balaenoptera* species with similarly complex colour patterns, such as the Omura's whale (Cerchio and Yamada 2018). Attaining calf photo‐ID records more broadly would substantively improve mark/recapture‐based data for evaluations of cetacean longevity, demography, movement, and other life history parameters.

Recruitment and calf mortality rates of this population remain unknown; however, our findings reinforce the importance of the area around Ribbon Reef No. 10, in the northern GBR, as one of high ecological importance for dwarf minke whales. The repeated migration of ‘Rudi’ to the same location over multiple years aligns with the decades of individual resighting history from the northern GBR (Birtles et al. 2001, 2002; Sobtzick 2010; Barr 2023) and contributes to the growing body of evidence supporting the designation of this aggregation area as a Biologically Important Area (BIA) for this undescribed subspecies. More broadly, these findings represent the first direct evidence of natal wintering ground fidelity in dwarf minke whales globally, and the stability of colour patterns over a decade of sightings further supports the possible application of pattern‐based individual identification across the genus *Balaenoptera*.

## Author Contributions

**Claire E. Wouters:** conceptualization (lead), data curation (lead), formal analysis (lead), investigation (lead), methodology (equal), validation (equal), visualization (equal), writing – original draft (lead), writing – review and editing (equal). **Suzanne Hillcoat:** data curation (supporting), validation (equal), writing – review and editing (equal). **Naomi M. Gardiner:** conceptualization (supporting), methodology (supporting), supervision (supporting), validation (supporting), writing – review and editing (equal). **R. Alastair Birtles:** conceptualization (equal), data curation (equal), funding acquisition (equal), project administration (lead), supervision (equal), validation (equal), writing – review and editing (equal).

## Funding

This work was supported primarily by in‐kind and voluntary contributions.

## Ethics Statement

Field research has been conducted under a succession of Great Barrier Reef Marine Park Authority permits, Queensland Parks and Wildlife Services permits, and James Cook University Animal Ethics approvals since 2006, the latest being A2835 and G19/39617.1. This research did not receive any specific funding.

## Conflicts of Interest

The authors declare no conflicts of interest.

## Data Availability

All the data that support the fundings of this study are available in the material of this article.
